# Supplementary material for: Combinatorial Gene Expression Profiling of Serum HULC, HOTAIR, and UCA1 lncRNAs to Differentiate Hepatocellular Carcinoma from Liver Diseases: A Systematic Review and Meta-Analysis
Source: Int J Mol Sci. 2024 Jan 19;25(2):1258. doi: 10.3390/ijms25021258 (PMC10816616; doi:10.3390/ijms25021258)
Supplement: Supplementary file 1 [file ijms-25-01258-s001.zip › ijms-2819947-supplementary.pdf]

Table S1 PRISMA 2020 checklist

Section and Topic	Item #	Checklist item	Location where item is reported
**TITLE**			
Title	1	Identify the report as a systematic review.	Title page
**ABSTRACT**			
Abstract	2	See the PRISMA 2020 for Abstracts checklist.	Line 32
**INTRODUCTION**			
Rationale	3	Describe the rationale for the review in the context of existing knowledge.	Line 106
Objectives	4	Provide an explicit statement of the objective(s) or question(s) the review addresses.	Line 109
**METHODS**			
Eligibility criteria	5	Specify the inclusion and exclusion criteria for the review and how studies were grouped for the syntheses.	Line 134
Information sources	6	Specify all databases, registers, websites, organisations, reference lists and other sources searched or consulted to identify studies. Specify the date when each source was last searched or consulted.	Line 128
Search strategy	7	Present the full search strategies for all databases, registers and websites, including any filters and limits used.	Supplement table S2-4
Selection process	8	Specify the methods used to decide whether a study met the inclusion criteria of the review, including how many reviewers screened each record and each report retrieved, whether they worked independently, and if applicable, details of automation tools used in the process.	Line 132
Data collection process	9	Specify the methods used to collect data from reports, including how many reviewers collected data from each report, whether they worked independently, any processes for obtaining or confirming data from study investigators, and if applicable, details of automation tools used in the process.	Line 152
Data items	10a	List and define all outcomes for which data were sought. Specify whether all results that were compatible with each outcome domain in each study were sought (e.g. for all measures, time points, analyses), and if not, the methods used to decide which results to collect.	Line 142
	10b	List and define all other variables for which data were sought (e.g. participant and intervention characteristics, funding sources). Describe any assumptions made about any missing or unclear information.	Line 154
Study risk of bias assessment	11	Specify the methods used to assess risk of bias in the included studies, including details of the tool(s) used, how many reviewers assessed each study and whether they worked independently, and if applicable, details of automation tools used in the process.	Line 164
Effect measures	12	Specify for each outcome the effect measure(s) (e.g. risk ratio, mean difference) used in the synthesis or presentation of results.	Line 175

Section and Topic	Item #	Checklist item	Location where item is reported
Synthesis methods	13a	Describe the processes used to decide which studies were eligible for each synthesis (e.g. tabulating the study intervention characteristics and comparing against the planned groups for each synthesis (item #5)).	Line 134
	13b	Describe any methods required to prepare the data for presentation or synthesis, such as handling of missing summary statistics, or data conversions.	Line 157
	13c	Describe any methods used to tabulate or visually display results of individual studies and syntheses.	Line 167
	13d	Describe any methods used to synthesize results and provide a rationale for the choice(s). If meta-analysis was performed, describe the model(s), method(s) to identify the presence and extent of statistical heterogeneity, and software package(s) used.	Line 176
	13e	Describe any methods used to explore possible causes of heterogeneity among study results (e.g. subgroup analysis, meta-regression).	Line 178
	13f	Describe any sensitivity analyses conducted to assess robustness of the synthesized results.	
Reporting bias assessment	14	Describe any methods used to assess risk of bias due to missing results in a synthesis (arising from reporting biases).	Line 184
Certainty assessment	15	Describe any methods used to assess certainty (or confidence) in the body of evidence for an outcome.	NA
**RESULTS**			
Study selection	16a	Describe the results of the search and selection process, from the number of records identified in the search to the number of studies included in the review, ideally using a flow diagram.	Line 188
	16b	Cite studies that might appear to meet the inclusion criteria, but which were excluded, and explain why they were excluded.	Supplement table S5
Study characteristics	17	Cite each included study and present its characteristics.	Supplement table S6
Risk of bias in studies	18	Present assessments of risk of bias for each included study.	Supplement table S8
Results of individual studies	19	For all outcomes, present, for each study: (a) summary statistics for each group (where appropriate) and (b) an effect estimate and its precision (e.g. confidence/credible interval), ideally using structured tables or plots.	Figure 3
Results of syntheses	20a	For each synthesis, briefly summarise the characteristics and risk of bias among contributing studies.	Table 1, Figure 2
	20b	Present results of all statistical syntheses conducted. If meta-analysis was done, present for each the summary estimate and its precision (e.g. confidence/credible interval) and measures of statistical	Figure 3-5

Section and Topic	Item #	Checklist item	Location where item is reported
		heterogeneity. If comparing groups, describe the direction of the effect.	
	20c	Present results of all investigations of possible causes of heterogeneity among study results.	NA
	20d	Present results of all sensitivity analyses conducted to assess the robustness of the synthesized results.	
Reporting biases	21	Present assessments of risk of bias due to missing results (arising from reporting biases) for each synthesis assessed.	NA
Certainty of evidence	22	Present assessments of certainty (or confidence) in the body of evidence for each outcome assessed.	NA
**DISCUSSION**			
Discussion	23a	Provide a general interpretation of the results in the context of other evidence.	Line 296
	23b	Discuss any limitations of the evidence included in the review.	Line 354
	23c	Discuss any limitations of the review processes used.	Line 361
	23d	Discuss implications of the results for practice, policy, and future research.	Line 380
**OTHER INFORMATION**			
Registration and protocol	24a	Provide registration information for the review, including register name and registration number, or state that the review was not registered.	Line 118
	24b	Indicate where the review protocol can be accessed, or state that a protocol was not prepared.	Line 118
	24c	Describe and explain any amendments to information provided at registration or in the protocol.	NA
Support	25	Describe sources of financial or non-financial support for the review, and the role of the funders or sponsors in the review.	Funding section
Competing interests	26	Declare any competing interests of review authors.	Declare section
Availability of data, code and other materials	27	Report which of the following are publicly available and where they can be found: template data collection forms; data extracted from included studies; data used for all analyses; analytic code; any other materials used in the review.	Cite as Supplement

From: Page MJ, McKenzie JE, Bossuyt PM, Boutron I, Hoffmann TC, Mulrow CD, et al. The PRISMA 2020 statement: an updated guideline for reporting systematic reviews. BMJ 2021;372:n71.

Table S2 Searching strategies: PubMed (December 7, 2022)

Database	Step	Search algorithm	Items found
**Pubmed**			
MeSH	#1	Liver neoplasm	188,262
	#2	Adenoma, liver cell	1,011
	#3	Carcinoma, hepatocellular	100,950
	#4	Liquid biopsy	11,024
	#5	RNA, long noncoding	30,400
	#6	#1 OR #2 OR #3 OR #4 OR #5	227,757
Domain / population	#7	Liver cancer	312,791
	#8	Hepatocellular carcinoma	141,317
	#9	Liver cell carcinoma	156,892
	#10	#7 OR #8 OR #9	341,629
Determinants	#11	Long untranslated rna	45,519
	#12	Long noncoding rna	43,054
	#13	lncRNA	48,219
	#14	#11 OR #12 OR #13	50,674
Procedures	#15	Circulat*	703,005
	#16	Plasma	1,028,936
	#17	serum	1,243,795
	#18	blood	5,363,064
	#19	urine	410,823
	#20	Bile	157,903
	#21	Non invasive testing	31,586
	#22	biopsy	4,196129
	#23	#15 OR #16 OR #17 OR #18 OR #19 OR #20 OR #21 OR #22	9,824,962
Study	#24	Prognos*	1,048,289
	#25	Diagnos*	5,959,438
	#26	association	5,613,472
	#27	Predicti*	1,069,090
	#28	#24 OR #25 OR #26 OR #27	10,998,184
Combine	#31	#6 AND #10 AND #14 AND #23 AND #28	1,262

Table S3 Searching strategies: EMBASE (December 7, 2022)

Database	Step	Search algorithm	Items found
**EMBASE**			
Domain / population	#1	Liver cancer	563,739
	#2	Hepatocellular carcinoma	166,834
	#3	Liver cell carcinoma	197,398
	#4	#1 OR #2 OR #3	579,806
Determinants	#5	Long untranslated rna	47,695
	#6	Long noncoding rna	44,612
	#7	lncRNA	32,353
	#8	#5 OR #6 OR #7	56,793
Procedures	#9	Circulat*	931,373
	#10	Plasma	1,410,541
	#11	serum	1,652,776
	#12	blood	5,936,537
	#13	urine	608,758
	#14	Bile	256,129
	#15	Non invasive testing	19,668
	#16	biopsy	1,030,861
	#17	#9 OR #10 OR #11 OR #12 OR #13 OR #14 OR #15 OR #16	8,790,301
Study	#18	Prognos*	1,404,636
	#19	Diagnos*	7,856,633
	#20	association	5,001,702
	#21	Predicti*	1,514,374
	#22	#18 OR #19 OR #20 OR #21	12,907,694
Combine	#23	#4 AND #8 AND #17 AND #22	725

Table S4 Searching strategies: Scopus (December 8, 2022)

Database	Step	Search algorithm	Items found
**Scopus**			
Domain	#1	(Hepatocellular AND carcinoma)	585,528
	#2	(human AND liver AND cancer)	1,375,022
Determinants	#3	(long AND noncoding AND RNA) OR (lncRNA) OR (untranslated AND rna)	245,955
Procedures	#4	(circulating AND rna) OR (liquid AND biopsy) OR (non AND invasive AND biopsy)	368,724
study	#5	(diagnosis) OR (prognosis)	8,612,372
	#6	#1 AND #2 AND #3 AND #4	6,123
	#7	Article only	2,638
	#8	English only	2,596

Table S5 Articles that were unretrievable and excluded

ID	Author	Year	Title	Journal	Retrieve (Y/N)	Include/Exclude	Reasons for exclusion	
P_001	Yang Z	2011	Overexpression of long non-coding RNA HOTAIR predicts tumor recurrence in hepatocellular carcinoma patients following liver transplantation	Ann Surg Oncol	Y	Excluded	1	tissue
P_002	Yuan SX	2012	Long noncoding RNA associated with microvascular invasion in hepatocellular carcinoma promotes angiogenesis and serves as a predictor for hepatocellular carcinoma patients' poor recurrence-free survival after hepatectomy	Hepatology	Y	Excluded	1	tissue, no control
P_006	Quagliata L	2015	HOTTIP expression is associated with metastasis formation, predicts outcome and is altered in plasma samples of hepatocellular carcinoma patients	Clinical and Experimental Metastasis	Y	Excluded	1	tissue
P_009	Yan IK	2015	Identification of candidate extracellular non-coding RNA biomarkers for Hepatocellular Carcinoma	Hepatology	Y	Excluded	6	Poster_384
P_012	Fu N	2016	Role of LncRNA-activated by transforming growth factor beta in the progression of hepatitis C virus-related liver fibrosis	Discov Med	Y	Excluded	1	HCV, not HCC
P_018	Zhuang LK	2016	MicroRNA-92b promotes hepatocellular carcinoma progression by targeting Smad7 and is mediated by long non-coding RNA XIST	Cell Death Dis	Y	Excluded	1	miRNA mechanism
P_021	Fang C	2017	Long non-coding ribonucleic acid zinc finger antisense 1 promotes the progression of colonic cancer by modulating ZEB1 expression	J Gastroenterol Hepatol	Y	Excluded	1	Colon Cancer
P_022	Liu Z	2017	Long non-coding RNA NEAT1 overexpression is associated with unfavorable prognosis in patients with hepatocellular carcinoma after hepatectomy: A Chinese population-based study	Eur J Surg Oncol	Y	Excluded	1	Tissue
P_025	Wang C	2017	Identification of long non-coding RNA p34822 as a potential plasma biomarker for the diagnosis of hepatocellular carcinoma	Sci China Life Sci	Y	Excluded	11	Letter to editor
P_032	Hu ML	2018	TGF-β1 upregulates the expression of lncRNA UCA1 and its downstream HXK2 to promote the growth of hepatocellular carcinoma	Eur Rev Med Pharmacol Sci	Y	Excluded	5	No raw data available, only KM plot
P_037	Topel H	2018	HGF/C-met signalling pathway downregulates lncrna hotair to induce adhesion independent growth in HCC by increasing caveolin-1 expression	ESMO Open	Y	Excluded	6	Poster
P_042	Yu ML	2018	Identification of circulating exosomal long non-coding RNA, DANCR, as liquid biopsy for prognostic markers of	Hepatology	Y	Excluded	6	Oral present abstract

ID	Author	Year	Title	Journal	Retrieve (Y/N)	Include/Exclude	Reasons for exclusion	
			hepatitis C virus-related hepatocellular carcinoma after surgical resection					
P_044	Zhang C	2018	lncRNA-HEIH in serum and exosomes as a potential biomarker in the HCV-related hepatocellular carcinoma	Cancer Biomark	Y	Excluded	2	no ROC, only relative expression level in graph
P_047	Burerina O	2019	Novel long non-coding RNA as a potential biomarker of liver cancer	FEBS Open Bio	Y	Excluded	6	Conference abstract
P_050	De los Angel Rojas	2019	Droplet digital pcr analysis of circulating lncrna H19: A potential biomarker for hepatocellular carcinoma	Hepatology	Y	Excluded	6	Poster 2040
P_054	Ma J	2019	A Noninvasive Prediction Nomogram for Lymph Node Metastasis of Hepatocellular Carcinoma Based on Serum Long Noncoding RNAs	Biomed Res Int	Y	Excluded	1	lymph node metastasis vs non-lymph node metastasis HCC
P_073	Niu JZ	2020	Long non-coding RNA Linc00261 as a novel potential diagnostic and prognostic biomarker for gallbladder cancer	Translational Cancer Research	Y	Excluded	11	Gall bladder CA
P_075	Rojas A	2020	LncRNA-H19 as a marker of liver progression from steatosis to hepatocellular carcinoma	Journal of Hepatology	Y	Excluded	6	Poster
P_079	Wang D	2020	Exosomal lncRNA H19 promotes the progression of hepatocellular carcinoma treated with Propofol via miR-520a-3p/LIMK1 axis	Cancer Med	Y	Excluded	1	Cell line
P_080	Wang SC	2020	Circulating exosome DANCR correlated to the recurrence of hepatitis C virus-related hepatocellular carcinoma	Journal of Clinical Oncology	Y	Excluded	6	Meeting abstract
P_081	Xia Q	2020	Identification and Analysis of the Blood lncRNA Signature for Liver Cirrhosis and Hepatocellular Carcinoma	Frontiers in Genetics	Y	Excluded	1	Data from gene expression omnibus, machine learning analysis
P_083	Fang Z	2021	Genome-wide long noncoding RNA and mRNA expression profiles demonstrate associations between exposure to inorganic elements and the risk of developing hepatocellular carcinoma	BMC Med Genomics	Y	Excluded	1	No validation of study
P_085	Guo Y	2021	Epigenetically-regulated serum GAS5 as a potential biomarker for patients with chronic hepatitis B virus infection	Cancer Biomark	Y	Excluded	1	Not HCC
P_087	Lee YR	2021	Circulating exosomal lncrna-atb promotes myopenia in human hepatocellular carcinoma	Hepatology	Y	Excluded	2	No comparison, Prognosis

ID	Author	Year	Title	Journal	Retrieve (Y/N)	Include/Exclude	Reasons for exclusion	
P_088	Mangan elli M	2021	10P Emerging role of Telomeric repeat-containing RNA TERRA in hepatocellular carcinoma	Annals of Oncology	Y	Excluded	6	Poster
P_092	Zhang L	2021	Serum lnc34a is a potential prediction biomarker for bone metastasis in hepatocellular carcinoma patients	BMC Cancer	Y	Excluded	1	HCC with bone metastasis compare to non met
P_094	Zhu Y	2021	Integrative analysis of long extracellular RNAs reveals a detection panel of noncoding RNAs for liver cancer	Theranostics	Y	Excluded	7	Too few patients information
P_102	Han C	2022	The expression of long non-coding RNA HOTAIR in advanced hepatocellular carcinoma and its prognostic correlation with sunitinib therapy	Archives of Medical Science	Y	Excluded	1	PBMC
P_105	Kunadir ek M	2022	Transcriptomic Analyses Reveal Long Non-Coding RNA in Peripheral Blood Mononuclear Cells as a Novel Biomarker for Diagnosis and Prognosis of Hepatocellular Carcinoma	Int J Mol Sci	Y	Excluded	1	PBMC
P_104	Huang XL	2022	Serum exosomal long noncoding RNA CRNDE level for hepatocellular carcinoma diagnosis	J Clin Lab Anal	Y	Excluded	11	Letter to editor
P_106	Li L	2022	Serum Exosomal lncRNA AC007099.1 Regulates the Expression of Neuropeptide-Related FAP, as a Potential Biomarker for Hepatocarcinogenesis	Dis Markers	Y	Excluded	10	Not primary data

Table S6 Characteristics of all eligible studies

study	Author	country	lncrna	types of sample	measurement method	n_case	case definition	n_control	control definition	References
P_003	Xie, 2014	China	HULC	Plasma	qRT-PCR	30	HCC	20	Healthy with no HCC and HBV	[1]
P_004	Li, 2015	China	HULC, Linc00152, UCA1, TUG1, CCAT1, MEG3, MALAT1, GAS5	Plasma	qRT-PCR	24	HCC	24	Healthy control	[2]
P_004_validation	Li, 2015	China	HULC; Linc00152	Plasma	qRT-PCR	66	HCC	53	Healthy control	[2]
P_005	Lu, 2015	China	lncRNAuc003wbd; lncRNA-AF085935	Serum	qRT-PCR	137	HCC	104 HBV; 138 HC	Hepatitis B infection; Healthy control who had negative HCC and HBV	[3]
P_007	Tang, 2015	China	RP11–160H22.5; XLOC_014172; LOC149086	Plasma	Microarray for screening; qRT-PCR in validation	Screen: 20; Validate: 147	HCC	Screen: 20; Validate: 180	Cancer free control	[4]
P_008	Wang, 2015	China	uc001ncr; AX800134	Serum	Microarray for screening (tumors); qRT-PCR in validation in blood	50	HBV-positiveHCC in validation	50 HBV; 50 HC	Hepatitis B infection; Healthy control Both in validation	[5]
P_010	El-Tawdi, 2015	Egypt	UCA1	Serum	qRT-PCR	70	HCC	32 HCV; 38 HC	Hepatitis C infection; Healthy control	[6]
P_011	El-Tawdi, 2016	Egypt	lncRNA-C terminal binding protein, androgen responsive (lncRNA–CTBP)	Serum	qRT-PCR	78	HCC	36 CHC; 44 HC	Chronic hepatitis cirrhosis; Healthy control	[7]
P_013	Huang, 2016	China	DGCR5	Serum	qRT-PCR	60	HCC	60	Healthy Control	[8]
P_014	Jing, 2016	China	SPRY4	Plasma	qRT-PCR	HCC pre-op, 60; HCC post-op, 60;	HCC at pre-operation; HCC at 2 week post-operation;	85 HBV; 63 HC	HBV &cirrhosis; Controls without hepatitis, hepatic disease and no abnormal liver biochemistry	[9]
P_015	Kamel, 2016	Egypt	lncRNA-UCA1; lncRNA-WRAP53	Serum	qRT-PCR	82	HCC	34 HCV; 44 HC	Chronic HCV infection; Healthy controls with no HCV, HBV infections or HCC	[10]

study	Author	country	lncrna	types of sample	measurement method	n_case	case definition	n_control	control definition	References
P_016	Ma, 2016	China	DANCR	Plasma	qRT-PCR	52	HCC	29 HBV;<br>22 LC;<br>43 HC	Hepatitis B infection;<br>Liver cirrhosis;<br>Healthy Control	[11]
P_017	Yu, 2016	China	PVT1;<br>uc002mbe.2	Serum	qRT-PCR	40	HCC	33	Healthy Control	[12]
P_019	Cao, 2017	China	UBE2CP3	Serum	qRT-PCR	pre-op, 40<br>post-op, 40	HCC at pre-operative and post-operative	75	Healthy volunteer	[13]
P_020	Chen, 2017	China	UCA1	Plasma	qRT-PCR	20 HCC with metastasis;<br>20 HCC without metastasis	HCC with intra-hepatic metastasis; HCC without metastasis	20	Healthy control	[14]
P_023	Ma, 2017	China	JPX;<br>XIST have no data and not significant	Plasma	qRT-PCR	42	HCC	68	Healthy controls without malignancies or HBV infection	[15]
P_024	Qin, 2017	China	BANCR		qRT-PCR	110	HCC	90 BLD;<br>120 HC	Benign liverdisease contaning chronic hepatitis, fatty liver and alcoholic liver disease;<br>Healthy control	[16]
P_026	Yuan, 2017	China	LINC00152, RP11-160H22.5, XLOC014172;LOC149086	Plasma	qRT-PCR	100	HCC	100 CH;<br>100 HC	Chronic hepatitis;<br>Healthy control	[17]
P_027	Abd El Gwad,2018	Egypt	LncRNA RP11-513I15.6	Serum	qRT-PCR	60	HCC	42 HCV;<br>18 HC	Hepatitis C infection;<br>Healthy control	[18]
P_029	Gao, 2018	China	SHNG1	Plasma	qRT-PCR	72	HCC	50 HBV;<br>50 HC	HBV-positive chronic hepatitis and cirrhosis;<br>Healthy control	[19]
P_030	Gong, 2018	China	nc-HOXC8-143	Plasma	qRT-PCR	200	HCC	200	Cancer free control	[20]
P_031	Gramantieri, 2018	Italy	CASC9	serum from fasting blood	qRT-PCR	14	Cirrhosis with HCC	10	Cirrhosis without HCC	[21]
P_033	Huang, 2018	China	linc-ITGB1	Serum	qRT-PCR	80	HCC	44	Healthy control	[22]
P_034	Li, 2018	China	FAL1	Serum (Exo)	qRT-PCR	30	HCC	30	Healthy control	[23]
P_035	Lou, 2018	China	ZFAS1	Plasma	qRT-PCR	80	HCC	75 HBV;<br>99 HC	Chronic hepatitis B;<br>Healthy control	[24]

study	Author	country	lncrna	types of sample	measurement method	n_case	case definition	n_control	control definition	References
P_036	Sun, 2018	China	LINC00161	Serum	qRT-PCR	20	HCC	20	Healthy control	[25]
P_036_ Val	Sun, 2018	China	LINC00162	Serum	qRT-PCR	56	HCC	56	Healthy control	[25]
P_038	Wang, 2018	China	GAS5-AS1	Plasma	qRT-PCR	63	HCC	46 HBV;<br>47 LC;<br>58 HC	Chronic hepatitis B;<br>Liver cirrhosis;<br>Healthy control	[26]
P_039	Wang, 2018	China	LRB1	Serum	qRT-PCR	326	HCC	73	Healthy control	[27]
P_040	Xie, 2018	China	lnc-PCDH9-13:1	plasma+saliva	qRT-PCR	10+10	HCC plasma+saliva samples	10+10	Healthy control plasma+saliva samples	[28]
P_040_ Val	Xie, 2018	China	lnc-PCDH9-13:2	saliva	qRT-PCR	50 early HCC;<br>50 advanced HCC	Early HCC (stage 1-2);<br>Advanced HCC (stage 3-4)	50 LC;<br>50 CHB;<br>50 HBsAG+;<br>50 HC	Liver cirrhosis;<br>Chronic hepatitis B;<br>HBsAG carriers;<br>Healthy control	[28]
P_041	Xu, 2018	China	Exosome: ENSG00000258332.1 ; LINC00635	serum	qRT-PCR	60	HCC	96 HBV;<br>85 LC;<br>60 HC	Hepatitis B infection;<br>Liver cirrhosis;<br>Healthy control	[29]
P_043	Zeng, 2018	China	DQ786243	serum	qRT-PCR	50	HCC	30	Healthy control	[30]
P_045	Zhang, 2018	China	RP11-466I1.1	serum	qRT-PCR	72	HBV-related HCC	11	Hemangioma patients	[31]
P_046	Zheng, 2018	China	UCA1	serum	qRT-PCR	105	HCC	105 BLD;<br>105 HC	Benign liver disease;<br>Healthy control and	[32]
P_048	Cao, 2019	China	Exosome HULC	serum (Exo)	qRT-PCR	30	HCC	NA	Healthy control	[33]
P_049	Chao, 2019	China	lncRNA-D16366	serum	qRT-PCR	107	HCC	58 LD;<br>85 HC	Benign liver disease defined as alcoholic liver disease,HBV, fatty liver and alcoholic liver disease;<br>Healthy control	[34]
P_051	Dong, 2019	China	MEG3	serum	qRT-PCR	54	HCC	54	Healthy control	[35]
P_052	Habieb, 2019	Egypt	lncRNA-TSIX	serum	qRT-PCR	65	HCC	32	Healthy control	[36]
P_055	Motawi, 2019	Egypt	lncRNA-AF085935;<br>lncRNAuc003wbd	serum	qRT-PCR	70	HCC	70 HBV;<br>70 HC	Hepatitis B infection;<br>Healthy control	[37]
P_056	Qi, 2019	China	IGF2AS	serum	qRT-PCR	34	HCC with HBV cirrhosis	70	HBV cirrhosis	[38]

study	Author	country	lncrna	types of sample	measurement method	n_case	case definition	n_control	control definition	References
P_057	Refai, 2019	Egypt	TUG1;<br>CASC2	Plasma	qRT-PCR	30	HCC with HCV	20 HCV;<br>20 HC	Hepatitis C infection;<br>Healthy control	[39]
P_058	Shaker, 2019	Egypt	NEAT	Serum	qRT-PCR	36	HCC	36	Healthy control	[40]
P_060	Wu F, 2019	China	ZFAS1	serum	qRT-PCR	84	HCC	50 CH/BLD;<br>50 HC	chronic hepatitis and cirrhosis as benign liver disease;<br>Healthy control	[41]
P_061	Xu, 2019	China	LINC00978	serum	qRT-PCR	58	HCC	45 Benign;<br>49 HC	Benign liver disease;<br>Healthy control and	[42]
P_062	Zeng, 2019	China	CASC9	Serum	qRT-PCR	80	HCC	50	healthy volunteer	[43]
P_063	Zeng, 2019	China	X91348	Serum	qRT-PCR	107	HCC	82	Healthy control	[44]
P_064	Fang, 2020	China	MAGI2-AS3	Plasma	qRT-PCR	68	HCC	68	Healthy control	[45]
P_065	Huang J, 2020	China	HULC, MALAT1, LINC00152, PTTG3P, SPRY4-IT1, UBE2CP3, UCA1	Serum	qRT-PCR	129	HCC	93	Healthy control	[46]
P_66	Huang X, 2020	China	RP11-544D21.1, RP11-919I15.4, RP11-85G21.1	Plasma(Exo)	qRT-PCR	112	HCC	52HC	Healthy control	[47]
P_67	Jiang L, 2020	China	HAND2-AS1	Serum	qRT-PCR	44	HCC	38 HB;<br>32 HC	Hepatitis C infection;<br>Healthy control	[48]
P_68	Kim SS, 2020	Korea	LINC00853	Serum(EV)	qRT-PCR	100	HCC	102 HC	Healthy control	[49]
P_69	Li Y, 2020	China	NONHSAT053785	Serum	ddPCR	112	HCC	99 HC	Healthy control	[50]
P_70	Lu Y, 2020	China	ENSG00000248932.1, ENST00000440688.1, ENST00000457302.2	Plasma(Exo)	qRT-PCR	200	HCC	200 HC	Healthy control	[51]
P_71	Matboli M, 2020	Egypt	RP11-538F2.2	Serum	qRT-PCR	60	HCC	18 HC	Healthy control	[52]
P_72	Mohyeldeen M,2020	Egypt	NEAT1, TUG1	Serum	qRT-PCR	40	HCC	40 HCV;<br>20 HC	Hepatitis C infection;<br>Healthy control	[53]
P_74	Qin SJ, 2020	China	ST8SIA6-AS1	Serum	qRT-PCR	77	HCC	55	Healthy control	[54]
P_76	Rhosdy F,2020	Egypt	HOTAIR, HOTTIP	Serum	qRT-PCR	25	HCC	50 CLD;<br>25 HC	Chronic liver disease with or without cirrhosis;<br>Healthy control;	[55]

study	Author	country	lncrna	types of sample	measurement method	n_case	case definition	n_control	control definition	References
P_77	Shaker OG, 2020	Egypt	HOTAIR	Serum	qRT-PCR	50	HCC	50 HCV; 45 HC	Hepatitis C infection; Healthy control	[56]
P_078	Song W, 2020	China	PVT1	Serum	qRT-PCR	94	HCC with HBV cirrhosis	52	HBV cirrhosis	[57]
P_82	Yao Z., 2020	China	GPR89B-15, FAM72D-, FAM21A-2, EPC1-4, ZEB2-19	Serum(Exo)	qRT-PCR	45	HCC	45 LD; 45 LC; 45 HC;	Liver disease; Liver cirrhosis; Healthy control	[58]
P_86	Kim S., 2021	South Korea	DELOU2, HOTTIP, MALAT1, SNHG1	Serum(EV)	qRT-PCR	100	HCC	36	Healthy control	[59]
P_89	Tian Q., 2021	China	BACE1-AS	Serum	qRT-PCR	28	HCC	28	Healthy control	[60]
P_90	Wang T., 2021	China	CRNDE	Serum(Exo)	qRT-PCR	166	HCC	100	Healthy control	[61]
P_91	You L., 2021	China	LINC00161	Serum(Exo)	qRT-PCR	56	HCC	56	Healthy control	[62]
P_93	Zhu X., 2021	China	LINC00485	Serum	qRT-PCR	70	HCC	70 Hepatitis/LC ; 70 HC	Hepatitis or liver cirrhosis; Healthy control	[63]
P_95	Ali M.A., 2022	Egypt	NBAT1, FOXCUT	Serum	qRT-PCR	165	HCC on top HCV	180 HCV; 180 HC	Hepatitis C infection with no proceding therapy for HCV; Healthy control	[64]
P_96	Bongolo C.C., 2022	China	LIPCAR	Plasma	qRT-PCR	70	HCC	96 LC/CHB; 64 HC	Liver cirrhosis and hepatitis B; Healthy control	[65]
P_97	Chen J., 2022	China	LINC00941, LINC00514	Serum	qRT-PCR	40	HCC	40 LC; 37CHB; 30 HC	Liver cirrhosis; Chronic hepatitis B; Healthy control male	[66]
P_98	Chen Y., 2022	China	PCNAP1	Plasma	qRT-PCR	127	HCC	127	Healthy control	[67]
P_99	El-Shendidi A., 2022	Egypt	HOTAIR	Serum	qRT-PCR	80 (40, stage C-D; 40, stage 0/A-B)	HCC on top HCV-LC, half of them were patients with tumor stages C-D according to the Barcelona Clinic Liver Cancer (BCLC) and the other half	20	Healthy control with no evidence of LD	[68]

study	Author	country	lncrna	types of sample	measurement method	n_case	case definition	n_control	control definition	References
							with tumor stages 0/A-B			
P_100	Fu P., 2022	China	AC005332.5, ELF3-AS1, LINC00665	Serum	qRT-PCR	86	HBV-related HCC	46	Healthy control	[69]
P_101	Gong A., 2022	China	C10orf91, LINC01224	Serum	qRT-PCR	70	HCC	50	Healthy control	[70]
P_103	Han Y., 2022	China	SCARNA10	Serum	qRT-PCR	127	HCC	55 BLD; 99 HC	Benign liver disease; Healthy control	[71]
P_107	Lou Z., 2022	China	HOTAIR, BRM, ICR	Serum	qRT-PCR	61	HCC	20 LC; 20 HC	Healthy control	[72]
P_108	Manganelli M., 2022	Italy	TERRA1, TERRA3, TERC	Plasma	qRT-PCR	25	HCC	25	Healthy control	[73]
P_109	Mo C., 2022	China	LINC01973	Whole blood	qRT-PCR	52	HCC-HBV positive	30 LC; 30 CHB; 30 HC	Liver cirrhosis; Chronic hepatitis B; Healthy control male	[74]
P_110	Mohammed S.R., 2022	Egypt	MEG3	Serum	qRT-PCR	114	HCC without treatment	110	Healthy control	[75]
P_111	Yao J, 2022	China	H19-204, THEMIS2-211, PRKACA-202	Plasma (Exo)	qRT-PCR	33	HCC	15	Healthy control	[76]
P_111_ Val	Yao J, 2022	China	H19-204, THEMIS2-211, PRKACA-203	Plasma (Exo)	qRT-PCR	59	HCC	41	Healthy control	[76]

Table S7 lncRNAs that have been studied in HCC, excluding PBMC

No.	lncRNAs	Publications	Author, Year [References]
1	UCA1	5	Li J., 2015; Kamel M.M., 2016; Chen C., 2017; Zheng Z., 2018; Huang J., 2020 [2, 10, 14, 32, 46]
2	HOTAIR	4	Rhosdy F., 2020; Shaker O.G., 2020; El-Shendidi A., 2022; Lou Z., 2022 [55, 56, 68, 72]
3	HULC	4	Xie H., 2014; Li J., 2015; Li J., 2015 (validation) Huang J., 2020 [1, 2, 46]
4	MALAT1	3	Li J., 2015; Huang J., 2020; Kim S., 2021 [2, 46, 59]
5	MEG3	3	Li J., 2015; Dong H., 2019; Mohammed S.R., 2022 [2, 35, 75]
6	TUG1	3	Li J., 2015; Refai N.S., 2019; Mohyeldeen M.,2020 [2, 39, 53]
7	AF085935	2	Lu J., 2015; Motawi T., 2019 [2, 37]
8	CASC9	2	Gramantieri L., 2018; Zeng Y., 2019 [21, 43]
9	GAS5	2	Li J., 2015; Wang Y., 2018 [2, 26]
10	HOTTIP	2	Rhosdy F., 2020; Kim S., 2021 [55, 59]
11	Linc00152	2	Li J., 2015; Huang J., 2020 [2, 46]
12	Linc00161	2	Sun L., 2018; You L., 2021 [25, 62]
13	lncRNA-AF085935	2	Lu J., 2015; Motawi T., 2019 [3, 37]
14	LOC149086	2	Tang J., 2015; Yuan Y., 2017 [4, 17]
15	NEAT	2	Shaker O.G., 2019; Mohyeldeen M.,2020 [40, 53]
16	RP11-160H22.5	2	Tang J., 2015; Yuan W., 2017 [4, 17]
17	SPRY4	2	Jing W., 2016; Huang J., 2020 [9, 46]
18	UBE2CP3	2	Cao S., 2017; Huang J., 2020 [13, 46]
19	uc003wbd	2	Lu J., 2015; Motawi T., 2019 [3, 37]
20	XLOC014172	2	Tang J., 2015; Yuan Y., 2017[4, 17]
21	ZFAS1	2	Lou P., 2018; Wu F., 2019 [24, 41]
22	AC005332.5	1	Fu P., 2022 [69]
23	BACE1-AS	1	Tian Q., 2021 [60]
24	BANCR	1	Qin Y., 2017 [16]
25	BRM	1	Lou Z., 2022 [72]
26	C10orf91	1	Gong A., 2022 [70]
27	CCAT	1	Li J., 2015 [2]
28	CRNDE	1	Wang T., 2021 [61]
29	CTBP	1	El-Tawdi A.H.F., 2016 [7]
30	DANCR	1	Ma X., 2016 [11]
31	DELOU2	1	Kim S., 2021 [59]

32	DGCR5	1	Huang R., 2016 [8]
33	DQ786243	1	Zeng B., 2018 [30]
34	ELF3-AS1	1	Fu P., 2022 [69]
35	ENSG00000248932.1	1	Lu Y., 2020 [51]
36	ENSG00000258332.1	1	Xu H., 2018 [29]
37	ENST00000440688.1	1	Lu Y., 2020 [51]
38	ENST00000457302.2	1	Lu Y., 2020 [51]
39	EPC1-4	1	Yao Z., 2020 [58]
40	FAL1	1	Li B., 2018 [23]
41	FAM21A-2	1	Yao Z., 2020 [58]
42	FAM72D	1	Yao Z, 2020 [58]
43	FOXCUT	1	Ali M.A., 2022 [64]
44	GPR89B-15	1	Yao Z., 2020 [58]
45	H19-204	1	Yao J., 2022 [58]
46	HAND2-AS1	1	Jiang L., 2020 [48]
47	ICR	1	Lou Z., 2022 [72]
48	IGF2AS	1	Qi J., 2019 [38]
49	ITGB1	1	Huang L., 2018 [22]
50	JPX	1	Ma W., 2017 [15]
51	LINC00152	1	Li J., 2015 [2]
52	LINC00485	1	Zhu X., 2021 [63]
53	LINC00514	1	Chen J., 2022 [66]
54	LINC00635	1	Xu H., 2018 [29]
55	LINC00665	1	Fu P., 2022 [69]
56	LINC00853	1	Kim S.S., 2020 [49]
57	LINC00941	1	Chen J., 2022 [66]
58	LINC00978	1	Xu X., 2019 [42]
59	LINC01224	1	Gong A., 2022 [70]
60	LINC01973	1	Mo C., 2022 [74]
61	LIPCAR	1	Bongolo C.C., 2022 [65]
62	lncRNA-D16366	1	Chao Y., 2019 [34]
63	lncRNA-TSIX	1	Habieb A., 2019 [36]
64	LRB1	1	Wang Z.F., 2018 [27]
65	MAGI2-AS3	1	Fang G., 2020 [45]
66	NBAT1	1	Ali M.A., 2022 [64]
67	nc-HOXC8-143	1	Gong L., 2018 [20]

68	NONHSAT053785	1	Li Y., 2020 [50]
69	PCDH9-13:1	1	Xie Z., 2018 [28]
70	PCNAP1	1	Chen Y., 2022 [67]
71	PRKACA-202	1	Yao J., 2022 [76]
72	PTTG3P	1	Huang J., 2020 [46]
73	PVT1	1	Yu J., 2016 [12]
74	RP11-466I1.1	1	Zhang J., 2018 [31]
75	RP11-513I15.6	1	Abd El Gwad,2018 [18]
76	RP11-538F2.2	1	Matboli M., 2020 [52]
77	RP11-544D21.1	1	Huang X., 2020 [46]
78	RP11-85G21.1	1	Huang X., 2020 [46]
79	RP11-919I15.4	1	Huang X., 2020 [46]
80	SCARNA10	1	Han Y., 2022 [71]
81	SHNG1	1	Gao S., 2018 [19]
82	TERC	1	Manganelli M., 2022 [73]
83	TERRA1	1	Manganelli M., 2022 [73]
84	TERRA3	1	Manganelli M., 2022 [73]
85	THEMIS2-211	1	Yao J., 2022 [76]
86	uc001ncr	1	Wang K., 2015 [5]
87	X91348	1	Zeng Z., 2019 [44]
88	ZEB2-19	1	Yao Z., 2020 [58]

Table S8.1 Risk of bias assessment of the candidate lncRNA studies

Study ID	P_003	Author	Xie et al., 2014	Assessor	
**Domain**	**Signalling question**			**Response**	**Comments**
**1. Patient selection**	1.1 Was a consecutive or random sample of patients enrolled?			Unclear	No information of case recruitment, exclusion criteria, with unmatched control
	1.2 Was a case-control design avoided?			-	
	1.3 Did the study avoid inappropriate exclusions?			Unclear	
	**Risk of bias judgement**			**Some concern**	**There is some concern in case recruitment since no diagnostic data provided.**
	1.4 Is there concern that the included patients do not match the review question?			No	HCC
	**Applicability judgement**			**Low risk**	**Match review question**
**2. Index test(s)**	2.1 Were the index test results interpreted without knowledge of the results of the reference standard?			Unclear	NI
	2.2 If a threshold was used, was it pre-specified?			No	No threshold for positive result, only % of detection.
	**Risk of bias judgement**			**Some concern**	**No crude expression level reported.**
	2.3 Is there concern that the index test, its conduct, or interpretation differ from the review question?			Yes	**No crude expression level reported.**
	**Applicability judgement**			**Some concern**	**No crude expression level reported.**
**3. Reference standard**	3.1 Is the reference standard likely to correctly classify the target condition?			Unclear	NI
	3.2 Were the reference standard results interpreted without knowledge of the results of the index test?			Unclear	NI
	**Risk of bias judgement**			**Some concern**	**No HCC diagnostic procedure specified.**
	3.3 Is there concern that the target condition as defined by the reference standard does not match the review question?			No	HCC
	**Applicability judgement**			**Low**	**Match review question, still contain some unclear HCC diagnosis.**
**4. Flow and timing**	4.1 Was there an appropriate interval between index test(s) and reference standard?			unclear	No information regarding the treatment before index test.
	4.2 Did all patients receive a reference standard?			Yes	
	4.3 Did patients receive the same reference standard?			unclear	No evidence of different reference standard
	4.4 Were all patients included in the analysis?			Yes	N analysed as recruited
	**Risk of bias judgement**			**Some concerns**	**Contains many unclear information which are crucial. Still, all patients were analysed, and no evidence of treatment before sample collection**

Table S8.2 Risk of bias assessment of the candidate lncRNA studies

Study ID	P_004	Author	Li et al., 2015	Assessor	
**Domain**	**Signalling question**			**Response**	**Comments**
**1. Patient selection**	1.1 Was a consecutive or random sample of patients enrolled?			Unclear	Case underwent hepatic resection; Controls were from healthy volunteers without any health problems. Unclear exclusion criteria.
	1.2 Was a case-control design avoided?			-	
	1.3 Did the study avoid inappropriate exclusions?			Unclear	
	**Risk of bias judgement**			**Low**	**Even unmatched case-control, clear case selection**
	1.4 Is there concern that the included patients do not match the review question?			**No**	HCC
	**Applicability judgement**			**Low**	**Match review question**
**2. Index test(s)**	2.1 Were the index test results interpreted without knowledge of the results of the reference standard?			Unclear	NI
	2.2 If a threshold was used, was it pre-specified?			No	No threshold
	**Risk of bias judgement**			**Low**	**Explore the fold change, then validate the results in new population. Still, no blinding specified.**
	2.3 Is there concern that the index test, its conduct, or interpretation differ from the review question?			**No**	lncRNA expression level
	**Applicability judgement**			**Low**	**Match review question**
**3. Reference standard**	3.1 Is the reference standard likely to correctly classify the target condition?			Yes	66 HCC patients underwent hepatic resections
	3.2 Were the reference standard results interpreted without knowledge of the results of the index test?			Unclear	No information
	**Risk of bias judgement**			**Low**	**All patients were diagnosed by histological examination.**
	3.3 Is there concern that the target condition as defined by the reference standard does not match the review question?			No	HCC
	**Applicability judgement**			**Low**	**Match review question**
**4. Flow and timing**	4.1 Was there an appropriate interval between index test(s) and reference standard?			unclear	No information
	4.2 Did all patients receive a reference standard?			Yes	
	4.3 Did patients receive the same reference standard?			Yes	All patients were diagnosed by histological examination.
	4.4 Were all patients included in the analysis?			Yes	N analysed as recruited
	**Risk of bias judgement**			**Some concerns**	**No evidence of treatment before index test.**

Table S8.3 Risk of bias assessment of the candidate lncRNA studies

Study ID	P_015	Author	Kamel et al, 2016	Assessor	
**Domain**	**Signalling question**			**Response**	**Comments**
**1. Patient selection**	1.1 Was a consecutive or random sample of patients enrolled?			Unclear	From January 2013 to February 2014, a total of 160 participants were enrolled into the study, assume consecutive. Healthy controls were excluded from the HCC, HCV diagnosis.
	1.2 Was a case-control design avoided?			-	
	1.3 Did the study avoid inappropriate exclusions?			Yes	
	**Risk of bias judgement**			**Low**	**Appropriate cases definition, though unmatched controls**
	1.4 Is there concern that the included patients do not match the review question?			**No**	
	**Applicability judgement**			**Low**	**Match the question**
**2. Index test(s)**	2.1 Were the index test results interpreted without knowledge of the results of the reference standard?			Unclear	NI
	2.2 If a threshold was used, was it pre-specified?			No	No pre-specified threshold
	**Risk of bias judgement**			**Some concerns**	**Data driven threshold, not blind interpretation.**
	2.3 Is there concern that the index test, its conduct, or interpretation differ from the review question?			**No**	Use lncRNA
	**Applicability judgement**			**Low**	**Match the question**
**3. Reference standard**	3.1 Is the reference standard likely to correctly classify the target condition?			Yes	All patients were diagnosed according to American Association for the Study of Liver Diseases practice guidelines. HCC clinical stage was determined according to Barcelona Clinic Liver Cancer staging classification14 and Child-Pugh classification.
	3.2 Were the reference standard results interpreted without knowledge of the results of the index test?			Unclear	NI
	**Risk of bias judgement**			**Low**	**Diagnostic criteria was specified**
	3.3 Is there concern that the target condition as defined by the reference standard does not match the review question?			No	HCC
	**Applicability judgement**			**Low**	**Match the question**
**4. Flow and timing**	4.1 Was there an appropriate interval between index test(s) and reference standard?			unclear	No information
	4.2 Did all patients receive a reference standard?			Yes	
	4.3 Did patients receive the same reference standard?			No	Some had tissue biopsy
	4.4 Were all patients included in the analysis?			Yes	N=160 as recruited
	**Risk of bias judgement**			**Low**	**NI for treatment, all included in analysis**

Table S8.4 Risk of bias assessment of the candidate lncRNA studies

Study ID	P_020	Author	Chen et al., 2017	Assessor	
**Domain**	**Signalling question**			**Response**	**Comments**
**1. Patient selection**	1.1 Was a consecutive or random sample of patients enrolled?			Yes	Plasma samples of 20 patients confirmed by combined diagnosis of imaging examination and surgical pathology results who were diagnosed from January 2015 to January 2016 were collected. Healthy control samples were collected
	1.2 Was a case-control design avoided?			-	
	1.3 Did the study avoid inappropriate exclusions?			Unclear	
	**Risk of bias judgement**			**Low**	Appropriate cases, unmatched control
	1.4 Is there concern that the included patients do not match the review question?			No	HCC
	**Applicability judgement**			**Low**	**Match the question**
**2. Index test(s)**	2.1 Were the index test results interpreted without knowledge of the results of the reference standard?			Unclear	NI
	2.2 If a threshold was used, was it pre-specified?			No	No threshold
	**Risk of bias judgement**			**Low**	**No evidence to blind result interpretation, and no threshold applied. Only the expression level reported.**
	2.3 Is there concern that the index test, its conduct, or interpretation differ from the review question?			**No**	
	**Applicability judgement**			**Low**	**lncRNA expression level match the question**.
**3. Reference standard**	3.1 Is the reference standard likely to correctly classify the target condition?			Yes	All patients with liver cancer and intrahepatic metastasis were confirmed by combined diagnosis.
	3.2 Were the reference standard results interpreted without knowledge of the results of the index test?			Unclear	NI
	**Risk of bias judgement**			**Low**	**Clearly defined reference test**
	3.3 Is there concern that the target condition as defined by the reference standard does not match the review question?			No	HCC
	**Applicability judgement**			**Low**	**Match review question**
**4. Flow and timing**	4.1 Was there an appropriate interval between index test(s) and reference standard?			Yes	All patients had not received radiotherapy and chemotherapy, immunotherapy, interventional therapy and liver transplantation., No intervention before the index test.
	4.2 Did all patients receive a reference standard?			Yes	
	4.3 Did patients receive the same reference standard?			Yes	
	4.4 Were all patients included in the analysis?			Yes	N=60 as recruited.
	**Risk of bias judgement**			**Low**	**Same reference test, no treatment before index test and all included in analysis.**

Table S8.5 Risk of bias assessment of the candidate lncRNA studies

Study ID	P_046	Author	Zheng et al., 2018	Assessor	
**Domain**	**Signalling question**			**Response**	**Comments**
1. Patient selection	1.1 Was a consecutive or random sample of patients enrolled?			Unclear	From June 2008 to July 2012, a total of 105 patients with histologically confirmed HCC were included in this study; 105 healthy volunteers (both age- and sex-matched). Patients with a history of previous cancer were excluded.
	1.2 Was a case-control design avoided?			-	
	1.3 Did the study avoid inappropriate exclusions?			Yes	
	**Risk of bias judgement**			**Low**	**Clear case definition, matched controls.**
	1.4 Is there concern that the included patients do not match the review question?			No	-
	**Applicability judgement**			**Low**	**Match review question**
2. Index test(s)	2.1 Were the index test results interpreted without knowledge of the results of the reference standard?			Unclear	NI
	2.2 If a threshold was used, was it pre-specified?			No	Not pre-specified.
	**Risk of bias judgement**			**Some concerns**	**Assume no blinding result interpretation, and no threshold pre-specified.**
	2.3 Is there concern that the index test, its conduct, or interpretation differ from the review question?			No	lncRNA expression level, still generalizability is warranted.
	**Applicability judgement**			**Low**	**Match review question**
3. Reference standard	3.1 Is the reference standard likely to correctly classify the target condition?			Yes	Histologically confirmed HCC
	3.2 Were the reference standard results interpreted without knowledge of the results of the index test?			Unclear	
	**Risk of bias judgement**			**Low**	**Clear case definition**
	3.3 Is there concern that the target condition as defined by the reference standard does not match the review question?			No	
	**Applicability judgement**			**Low**	**Match review question**
4. Flow and timing	4.1 Was there an appropriate interval between index test(s) and reference standard?			Yes	Serum samples were drawn before surgery. No chemotherapy, radiotherapy, or targeted therapy was used prior to blood collection.
	4.2 Did all patients receive a reference standard?			Yes	-
	4.3 Did patients receive the same reference standard?			Yes	105 patients with histologically confirmed
	4.4 Were all patients included in the analysis?			Yes	N analysed as recruited.
	**Risk of bias judgement**			**Low**	**Same reference test, all included in analyses and no prior treatment.**

Table S8.6 Risk of bias assessment of the candidate lncRNA studies

Study ID	P_051	Author	Dong et al., 2019	Assessor	
**Domain**	**Signalling question**			**Response**	**Comments**
1. Patient selection	1.1 Was a consecutive or random sample of patients enrolled?			Unclear	Patients with pathological and imaging confirmation were **selected** from the HCC cases. Controls from the hospitals with no significant difference of age and sex.
	1.2 Was a case-control design avoided?			-	
	1.3 Did the study avoid inappropriate exclusions?			Unclear	
	**Risk of bias judgement**			**Low**	**Similar controls, though highly concern for patient selection.**
	1.4 Is there concern that the included patients do not match the review question?			No	-
	**Applicability judgement**			**Low**	**Match review question**
2. Index test(s)	2.1 Were the index test results interpreted without knowledge of the results of the reference standard?			Unclear	NI
	2.2 If a threshold was used, was it pre-specified?			No	No threshold applied
	**Risk of bias judgement**			**Some concerns**	**Not pre-specified cut-off and no clear evidence of blinding when interpret the results.**
	2.3 Is there concern that the index test, its conduct, or interpretation differ from the review question?			No	-
	**Applicability judgement**			**Low**	**Match review question**
3. Reference standard	3.1 Is the reference standard likely to correctly classify the target condition?			Yes	Pathological and imaging examinations were performed for all those patients
	3.2 Were the reference standard results interpreted without knowledge of the results of the index test?			Unclear	NI
	**Risk of bias judgement**			**Low**	**Clear diagnostic criteria**
	3.3 Is there concern that the target condition as defined by the reference standard does not match the review question?			No	-
	**Applicability judgement**			**Low**	**Match review question**
4. Flow and timing	4.1 Was there an appropriate interval between index test(s) and reference standard?			Unclear	NI of treatment prior to sample collection.
	4.2 Did all patients receive a reference standard?			Yes	All
	4.3 Did patients receive the same reference standard?			Yes	All received Pathological and imaging examinations.
	4.4 Were all patients included in the analysis?			Yes	N analysed as recruited.
	**Risk of bias judgement**			**Low**	**All received same reference test, and included to the analyses, though no information of prior treatment.**

Table S8.7 Risk of bias assessment of the candidate lncRNA studies

Study ID	P_057	Author	Refai et al., 2019	Assessor	
**Domain**	**Signalling question**			**Response**	**Comments**
**1. Patient selection**	1.1 Was a consecutive or random sample of patients enrolled?			Yes	30 newly diagnosed HCC (CHILD class A with no extrahepatic spread or lymph node involvement) on top of HCV patients according to AASLD Practice Guidelines. Unmatched healthy controls
	1.2 Was a case-control design avoided?			-	
	1.3 Did the study avoid inappropriate exclusions?			Yes	
	**Risk of bias judgement**			**Low**	**Unmatched healthy controls, but with clear case definition.**
	1.4 Is there concern that the included patients do not match the review question?			No	-
	**Applicability judgement**			**Low**	**Match review question**
**2. Index test(s)**	2.1 Were the index test results interpreted without knowledge of the results of the reference standard?			unclear	NI
	2.2 If a threshold was used, was it pre-specified?			No	Not pre-specified.
	**Risk of bias judgement**			**Some concerns**	**Not pre-specified cut-off and no clear evidence of blinding when interpret the results.**
	2.3 Is there concern that the index test, its conduct, or interpretation differ from the review question?			No	-
	**Applicability judgement**			**Low**	**Match review question**
**3. Reference standard**	3.1 Is the reference standard likely to correctly classify the target condition?			Yes	According to AASLD Practice Guidelines
	3.2 Were the reference standard results interpreted without knowledge of the results of the index test?			Unclear	NI
	**Risk of bias judgement**			**Low**	**Clear case definition.**
	3.3 Is there concern that the target condition as defined by the reference standard does not match the review question?			No	-
	**Applicability judgement**			**Low**	**Match review question**
**4. Flow and timing**	4.1 Was there an appropriate interval between index test(s) and reference standard?			Yes	Newly diagnosed patients who had no previous radiotherapy or systemic chemotherapy or had undergone liver transplantation.
	4.2 Did all patients receive a reference standard?			Yes	
	4.3 Did patients receive the same reference standard?			Yes	
	4.4 Were all patients included in the analysis?			Yes	
	**Risk of bias judgement**			**Low**	**All patients received same reference test and included in the analysis.**

Table S8.8 Risk of bias assessment of the candidate lncRNA studies

Study ID	P_065	Author	Huang et al., 2020	Assessor	
**Domain**	**Signalling question**			**Response**	**Comments**
**1. Patient selection**	1.1 Was a consecutive or random sample of patients enrolled?			Unclear	The patients with HCC were histologically confirmed. As a healthy control, 93 individuals who performed their annual health check at the hospital and did not have any liver diseases or other cancerous diseases were recruited.
	1.2 Was a case-control design avoided?			-	
	1.3 Did the study avoid inappropriate exclusions?			Yes	
	**Risk of bias judgement**			**Low**	**Unmatched control, but clear case definition.**
	1.4 Is there concern that the included patients do not match the review question?			No	-
	**Applicability judgement**			**Low**	**Match review question**
**2. Index test(s)**	2.1 Were the index test results interpreted without knowledge of the results of the reference standard?			Unclear	NI
	2.2 If a threshold was used, was it pre-specified?			No	Not pre-specified, even not reported
	**Risk of bias judgement**			**Some concerns**	**Not pre-specified threshold and no evidence of blinding in result interpretation.**
	2.3 Is there concern that the index test, its conduct, or interpretation differ from the review question?			No	-
	**Applicability judgement**			**Some concerns**	**Match review question, but no cut-off point for applicability and generalizability.**
**3. Reference standard**	3.1 Is the reference standard likely to correctly classify the target condition?			Yes	HCC were histologically confirmed.
	3.2 Were the reference standard results interpreted without knowledge of the results of the index test?			Unclear	NI
	**Risk of bias judgement**			**Low**	**Clear case definition.**
	3.3 Is there concern that the target condition as defined by the reference standard does not match the review question?			No	-
	**Applicability judgement**			**Low**	**Match review question**
**4. Flow and timing**	4.1 Was there an appropriate interval between index test(s) and reference standard?			Unclear	NI
	4.2 Did all patients receive a reference standard?			Yes	
	4.3 Did patients receive the same reference standard?			Yes	
	4.4 Were all patients included in the analysis?			Yes	
	**Risk of bias judgement**			Low	**All patients received same reference test, and all included in the analysis.**

Table S8.9 Risk of bias assessment of the candidate lncRNA studies

Study ID	P_072	Author	Mohyeldeen et al., 2020	Assessor	
**Domain**	**Signalling question**			**Response**	**Comments**
**1. Patient selection**	1.1 Was a consecutive or random sample of patients enrolled?			Unclear	HCC was diagnosed by the presence of liver focal lesions which were detected by ultrasound and further confirmed by CT or MRI according to the protocol of European Association of the Study of the Liver and were staged according to Barcelona Clinic Liver Cancer staging system. The age and sex of control volunteers were statistically checked to be matching those of the patient population. Exclusion criteria included a history HBV,HIV, previous therapy of HCC, HCV.
	1.2 Was a case-control design avoided?			-	
	1.3 Did the study avoid inappropriate exclusions?			Yes	
	**Risk of bias judgement**			**Low**	**Similar controls, clear case definition.**
	1.4 Is there concern that the included patients do not match the review question?			No	-
	**Applicability judgement**			**Low**	**Match review question**
**2. Index test(s)**	2.1 Were the index test results interpreted without knowledge of the results of the reference standard?			Unclear	NI
	2.2 If a threshold was used, was it pre-specified?			No	Not pre-specified
	**Risk of bias judgement**			**Some concerns**	**Not pre-specified threshold and no evidence of blinding in result interpretation.**
	2.3 Is there concern that the index test, its conduct, or interpretation differ from the review question?			No	-
	**Applicability judgement**			**Low**	**Match review question**
**3. Reference standard**	3.1 Is the reference standard likely to correctly classify the target condition?			Yes	clear case definition.
	3.2 Were the reference standard results interpreted without knowledge of the results of the index test?			Unclear	NI
	**Risk of bias judgement**			**Low**	**clear case definition.**
	3.3 Is there concern that the target condition as defined by the reference standard does not match the review question?			No	-
	**Applicability judgement**			**Low**	**Match review question**
**4. Flow and timing**	4.1 Was there an appropriate interval between index test(s) and reference standard?			Unclear	Patients with previous therapy of HCC, HCV were excluded.
	4.2 Did all patients receive a reference standard?			Yes	
	4.3 Did patients receive the same reference standard?			Yes	Assume same reference test
	4.4 Were all patients included in the analysis?			Yes	
	**Risk of bias judgement**			**Low**	**All patients received same reference test, and all included in the analysis.**

Table S8.10 Risk of bias assessment of the candidate lncRNA studies

Study ID	P_076	Author	Roshdy et al., 2020	Assessor	
**Domain**	**Signalling question**			**Response**	**Comments**
**1. Patient selection**	1.1 Was a consecutive or random sample of patients enrolled?			Unclear	Diagnosis of HCC was depending upon the presence of focal hepatic lesions diagnosed by abdominal ultrasound and confirmed by triphasic spiral CT and/or MRI according to the American Association for the Study of Liver Diseases (AASLD) 2011 guidelines. Exclusion criteria included any concomitant cause CLD, malignancies other than HCC. Age- and sex-matched healthy adults served as a control group
	1.2 Was a case-control design avoided?			-	
	1.3 Did the study avoid inappropriate exclusions?			Yes	
	**Risk of bias judgement**			**Low**	**Matched controls, clear case definition**
	1.4 Is there concern that the included patients do not match the review question?			No	-
	**Applicability judgement**			**Low**	**Match review question**
**2. Index test(s)**	2.1 Were the index test results interpreted without knowledge of the results of the reference standard?			Unclear	NI
	2.2 If a threshold was used, was it pre-specified?			No	Not pre-specified
	**Risk of bias judgement**			**Some concerns**	**Not pre-specified threshold and no evidence of blinding in result interpretation.**
	2.3 Is there concern that the index test, its conduct, or interpretation differ from the review question?			No	-
	**Applicability judgement**			**Low**	**Match review question**
**3. Reference standard**	3.1 Is the reference standard likely to correctly classify the target condition?			Yes	clear case definition.
	3.2 Were the reference standard results interpreted without knowledge of the results of the index test?			Unclear	NI
	**Risk of bias judgement**			**Low**	**clear case definition but the research question was on HCV-induced HCC.**
	3.3 Is there concern that the target condition as defined by the reference standard does not match the review question?			No	-
	**Applicability judgement**			**Low**	**Match review question**
**4. Flow and timing**	4.1 Was there an appropriate interval between index test(s) and reference standard?			Unclear	Patients did not receive any specific treatment for HCV during the last 6 months.
	4.2 Did all patients receive a reference standard?			Yes	
	4.3 Did patients receive the same reference standard?			Yes	
	4.4 Were all patients included in the analysis?			Yes	
	**Risk of bias judgement**			**Some concerns**	**All patients received same reference test, but no evidence of no HCC treatment before enrolment.**

Table S8.11 Risk of bias assessment of the candidate lncRNA studies

**Study ID**	P_077	**Author**	Shaker et al., 2020	**Assessor**	
**Domain**	**Signalling question**			**Response**	**Comments**
**1. Patient selection**	1.1 Was a consecutive or random sample of patients enrolled?			Unclear	50 HCC patients on top of HCV chronically infected Egyptian patients. 45 healthy volunteers serving as controls.
	1.2 Was a case-control design avoided?			-	
	1.3 Did the study avoid inappropriate exclusions?			No	
	**Risk of bias judgement**			**Some concerns**	Unclear case definition of HCC and unmatched controls
	1.4 Is there concern that the included patients do not match the review question?			**No**	-
	**Applicability judgement**			**Low**	**Still HCC, but render some unclear information.**
**2. Index test(s)**	2.1 Were the index test results interpreted without knowledge of the results of the reference standard?			Unclear	NI
	2.2 If a threshold was used, was it pre-specified?			No	Not pre-specified
	**Risk of bias judgement**			**Some concerns**	**Not pre-specified threshold and no evidence of blinding in result interpretation.**
	2.3 Is there concern that the index test, its conduct, or interpretation differ from the review question?			No	-
	**Applicability judgement**			**Low**	**Match review question**
**3. Reference standard**	3.1 Is the reference standard likely to correctly classify the target condition?			Unclear	Unclear diagnostic definition.
	3.2 Were the reference standard results interpreted without knowledge of the results of the index test?			Unclear	NI
	**Risk of bias judgement**			**Some concerns**	**Unclear diagnostic definition.**
	3.3 Is there concern that the target condition as defined by the reference standard does not match the review question?			No	-
	**Applicability judgement**			**Low**	**Match review question**
**4. Flow and timing**	4.1 Was there an appropriate interval between index test(s) and reference standard?			Unclear	NI
	4.2 Did all patients receive a reference standard?			Unclear	NI
	4.3 Did patients receive the same reference standard?			Unclear	NI
	4.4 Were all patients included in the analysis?			Unclear	Baseline demographic of control was not reported.
	**Risk of bias judgement**			**Some concerns**	**Some baseline was not reported, and no information of treatment received before blood collection.**

Table S8.12 Risk of bias assessment of the candidate lncRNA studies

Study ID	P_086	Author	Kim et al., 2021	Assessor	
**Domain**	**Signalling question**			**Response**	**Comments**
**1. Patient selection**	1.1 Was a consecutive or random sample of patients enrolled?			Unclear	HCC was diagnosed according to the American Association for the Study of Liver Diseases practice guideline. Healthy controls were defined as 18- to 50-year-old subjects with no past medical history who attended regular medical check-up
	1.2 Was a case-control design avoided?			-	
	1.3 Did the study avoid inappropriate exclusions?			No	
	**Risk of bias judgement**			**Low**	**Unmatched controls, clear case definition**
	1.4 Is there concern that the included patients do not match the review question?			No	-
	**Applicability judgement**			**Low**	**Match review question**
**2. Index test(s)**	2.1 Were the index test results interpreted without knowledge of the results of the reference standard?			Unclear	NI
	2.2 If a threshold was used, was it pre-specified?			No	Not pre-specified
	**Risk of bias judgement**			**Some concerns**	**Not pre-specified threshold and no evidence of blinding in result interpretation.**
	2.3 Is there concern that the index test, its conduct, or interpretation differ from the review question?			No	-
	**Applicability judgement**			**Low**	**Match review question**
**3. Reference standard**	3.1 Is the reference standard likely to correctly classify the target condition?			Yes	Clear diagnostic criteria.
	3.2 Were the reference standard results interpreted without knowledge of the results of the index test?			Unclear	NI
	**Risk of bias judgement**			**Low**	**Clear diagnostic criteria.**
	3.3 Is there concern that the target condition as defined by the reference standard does not match the review question?			No	-
	**Applicability judgement**			**Low**	**Match review question**
**4. Flow and timing**	4.1 Was there an appropriate interval between index test(s) and reference standard?			Unclear	NI
	4.2 Did all patients receive a reference standard?			Yes	Probably yes
	4.3 Did patients receive the same reference standard?			Yes	Probably yes
	4.4 Were all patients included in the analysis?			No	Some was reported.
	**Risk of bias judgement**			**Some concerns**	**Expression level was reported only in some cases, and no information of treatment received before blood collection.**

Table S8.13 Risk of bias assessment of the candidate lncRNA studies

Study ID	P_099	Author	El-Shendidi et al., 2022	Assessor	

Domain	Signalling question	Response	Comments
**1. Patient selection**	1.1 Was a consecutive or random sample of patients enrolled?	Unclear	Cases were newly diagnosed HCC on top of hepatitis C virus (HCV)-related liver cirrhosis, divided into tumor stages according to the Barcelona Clinic Liver Cancer Exclusion criteria were non-HCC hepatic malignancy, extra-hepatic malignancy, other causes of liver disease, and previous surgical, locoregional or systemic therapies of HCC. Healthy subjects with no evidence of liver disease were controls.
	1.2 Was a case-control design avoided?	-	
	1.3 Did the study avoid inappropriate exclusions?	Yes	
	**Risk of bias judgement**	**Low**	Unmatched controls, but clear definition of cases.
	1.4 Is there concern that the included patients do not match the review question?	No	-
	**Applicability judgement**	**Low**	**Match review question**
**2. Index test(s)**	2.1 Were the index test results interpreted without knowledge of the results of the reference standard?	Unclear	NI
	2.2 If a threshold was used, was it pre-specified?	No	Not pre-specified
	**Risk of bias judgement**	**Some concerns**	**Not pre-specified threshold and no evidence of blinding in result interpretation.**
	2.3 Is there concern that the index test, its conduct, or interpretation differ from the review question?	No	-
	**Applicability judgement**	**Low**	**Match review question**
**3. Reference standard**	3.1 Is the reference standard likely to correctly classify the target condition?	Yes	Clear diagnostic criteria. (The diagnosis of HCC was confirmed by triphasic CT and/or dynamic MRI).
	3.2 Were the reference standard results interpreted without knowledge of the results of the index test?	Unclear	NI
	**Risk of bias judgement**	**Low**	**Clear diagnostic criteria.**
	3.3 Is there concern that the target condition as defined by the reference standard does not match the review question?	No	-
	**Applicability judgement**	**Low**	**Match review question**
**4. Flow and timing**	4.1 Was there an appropriate interval between index test(s) and reference standard?	Yes	Cases included newly diagnosed HCC patients. Previous surgical, locoregional or systemic therapies of HCC were excluded.
	4.2 Did all patients receive a reference standard?	Yes	
	4.3 Did patients receive the same reference standard?	Yes	
	4.4 Were all patients included in the analysis?	Yes	N analysed as recruited.
	**Risk of bias judgement**	**Low**	**No treatment before blood collection and all samples were analysed.**

Table S8.14 Risk of bias assessment of the candidate lncRNA studies

Study ID	P_107	Author	Lou et al., 2022	Assessor	
**Domain**	**Signalling question**			**Response**	**Comments**
**1. Patient selection**	1.1 Was a consecutive or random sample of patients enrolled?			Unclear	Diagnosis of HCC was based on clinical symptoms, serum AFP levels, imaging and histology examination. Patients with a history of other tumours, and those receiving radiotherapy or chemotherapy were excluded from the study. Healthy subjects identified by physical examination
	1.2 Was a case-control design avoided?				
	1.3 Did the study avoid inappropriate exclusions?				
	**Risk of bias judgement**			**Low**	**Clear case definition, still unmatched controls.**
	1.4 Is there concern that the included patients do not match the review question?			No	-
	**Applicability judgement**			**Low**	**Match review question**
**2. Index test(s)**	2.1 Were the index test results interpreted without knowledge of the results of the reference standard?			Unclear	NI
	2.2 If a threshold was used, was it pre-specified?			No	Not pre-specified
	**Risk of bias judgement**			**Some concerns**	**Not pre-specified threshold and no evidence of blinding in result interpretation.**
	2.3 Is there concern that the index test, its conduct, or interpretation differ from the review question?			No	-
	**Applicability judgement**			**Low**	**Match review question**
**3. Reference standard**	3.1 Is the reference standard likely to correctly classify the target condition?			Yes	Diagnosis of HCC was based on clinical symptoms, serum AFP levels, imaging studies (ultrasound, CT, and MRI) and histopathological examination.
	3.2 Were the reference standard results interpreted without knowledge of the results of the index test?			Unclear	NI
	**Risk of bias judgement**			**Low**	**Clear diagnostic criteria.**
	3.3 Is there concern that the target condition as defined by the reference standard does not match the review question?			No	-
	**Applicability judgement**			**Low**	**Match review question**
**4. Flow and timing**	4.1 Was there an appropriate interval between index test(s) and reference standard?			Yes	Serum samples were collected from 34 HCC patients before and one week after surgery
	4.2 Did all patients receive a reference standard?			Yes	
	4.3 Did patients receive the same reference standard?			Yes	
	4.4 Were all patients included in the analysis?			Yes	N analysed as recruited.
	**Risk of bias judgement**			**Low**	**No treatment before blood collection, the after-treatment samples were analysed separately and all samples were analysed.**

Table S8.15 Risk of bias assessment of the candidate lncRNA studies

Study ID	P_065	Author	Mohammed et al., 2022	Assessor	
**Domain**	**Signalling question**			**Response**	**Comments**
**1. Patient selection**	1.1 Was a consecutive or random sample of patients enrolled?			Unclear	Diagnosis of the patients depended on history and imaging (CT, MRI). Patients were excluded if they had non-alcoholic steatohepatitis, malignancies other than HCC, diabetes, or autoimmune hepatitis. Control group consisted of 110 healthy individuals of matched age and sex with no history of cancer
	1.2 Was a case-control design avoided?			-	
	1.3 Did the study avoid inappropriate exclusions?			Yes	
	**Risk of bias judgement**			**Low**	**Matched control and very clear case definition.**
	1.4 Is there concern that the included patients do not match the review question?			No	-
	**Applicability judgement**			**Low**	**Match review question**
**2. Index test(s)**	2.1 Were the index test results interpreted without knowledge of the results of the reference standard?			Unclear	NI
	2.2 If a threshold was used, was it pre-specified?			No	Not pre-specified.
	**Risk of bias judgement**			**Some concerns**	Not pre-specified threshold and no blind of result interpretation.
	2.3 Is there concern that the index test, its conduct, or interpretation differ from the review question?			No	-
	**Applicability judgement**			**Low**	**Match review question**
**3. Reference standard**	3.1 Is the reference standard likely to correctly classify the target condition?			Yes	
	3.2 Were the reference standard results interpreted without knowledge of the results of the index test?			Unclear	NI
	**Risk of bias judgement**			Low	Clear case definition.
	3.3 Is there concern that the target condition as defined by the reference standard does not match the review question?			No	-
	**Applicability judgement**			**Low**	**Match review question**
**4. Flow and timing**	4.1 Was there an appropriate interval between index test(s) and reference standard?			Yes	All patients were newly diagnosed and had not received chemotherapy or radiotherapy treatment before enrolment.
	4.2 Did all patients receive a reference standard?			Yes	
	4.3 Did patients receive the same reference standard?			Yes	
	4.4 Were all patients included in the analysis?			Yes	
	**Risk of bias judgement**			**Low**	**All patients received same reference test, no prior treatment and included in the analysis.**

References

1. Xie, H.; Ma, H.; Zhou, D., Plasma HULC as a promising novel biomarker for the detection of hepatocellular carcinoma. *Biomed Res Int* **2013,** 2013, 136106.
2. Li, J.; Wang, X.; Tang, J.; Jiang, R.; Zhang, W.; Ji, J.; Sun, B., HULC and Linc00152 Act as Novel Biomarkers in Predicting Diagnosis of Hepatocellular Carcinoma. *Cell Physiol Biochem* **2015,** 37, (2), 687-96.
3. Lu, J.; Xie, F.; Geng, L.; Shen, W.; Sui, C.; Yang, J., Investigation of serum lncRNA-uc003wbd and lncRNA-AF085935 expression profile in patients with hepatocellular carcinoma and HBV. *Tumour Biol* **2015,** 36, (5), 3231-6.
4. Tang, J.; Jiang, R.; Deng, L.; Zhang, X.; Wang, K.; Sun, B., Circulation long non-coding RNAs act as biomarkers for predicting tumorigenesis and metastasis in hepatocellular carcinoma. *Oncotarget* **2015,** 6, (6), 4505-15.
5. Wang, K.; Guo, W. X.; Li, N.; Gao, C. F.; Shi, J.; Tang, Y. F.; Shen, F.; Wu, M. C.; Liu, S. R.; Cheng, S. Q., Serum LncRNAs Profiles Serve as Novel Potential Biomarkers for the Diagnosis of HBV-Positive Hepatocellular Carcinoma. *PLoS One* **2015,** 10, (12), e0144934.
6. El-Tawdi, A. H.; Matboli, M.; El-Nakeep, S.; Azazy, A. E.; Abdel-Rahman, O., Association of long noncoding RNA and c-JUN expression in hepatocellular carcinoma. *Expert Rev Gastroenterol Hepatol* **2016,** 10, (7), 869-77.
7. El-Tawdi, A. H.; Matboli, M.; Shehata, H. H.; Tash, F.; El-Khazragy, N.; Azazy Ael, S.; Abdel-Rahman, O., Evaluation of Circulatory RNA-Based Biomarker Panel in Hepatocellular Carcinoma. *Mol Diagn Ther* **2016,** 20, (3), 265-77.
8. Huang, R.; Wang, X.; Zhang, W.; Zhangyuan, G.; Jin, K.; Yu, W.; Xie, Y.; Xu, X.; Wang, H.; Sun, B., Down-Regulation of LncRNA DGCR5 Correlates with Poor Prognosis in Hepatocellular Carcinoma. *Cell Physiol Biochem* **2016,** 40, (3), 707-715.
9. Jing, W.; Gao, S.; Zhu, M.; Luo, P.; Jing, X.; Chai, H.; Tu, J., Potential diagnostic value of lncRNA SPRY4-IT1 in hepatocellular carcinoma. *Oncol Rep* **2016,** 36, (2), 1085-92.
10. Kamel, M. M.; Matboli, M.; Sallam, M.; Montasser, I. F.; Saad, A. S.; El-Tawdi, A. H. F., Investigation of long noncoding RNAs expression profile as potential serum biomarkers in patients with hepatocellular carcinoma. *Transl Res* **2016,** 168, 134-145.
11. Ma, X.; Wang, X.; Yang, C.; Wang, Z.; Han, B.; Wu, L.; Zhuang, L., DANCR Acts as a Diagnostic Biomarker and Promotes Tumor Growth and Metastasis in Hepatocellular Carcinoma. *Anticancer Res* **2016,** 36, (12), 6389-6398.
12. Yu, J.; Han, J.; Zhang, J.; Li, G.; Liu, H.; Cui, X.; Xu, Y.; Li, T.; Liu, J.; Wang, C., The long noncoding RNAs PVT1 and uc002mbe.2 in sera provide a new supplementary method for hepatocellular carcinoma diagnosis. *Medicine (Baltimore)* **2016,** 95, (31), e4436.
13. Cao, S. W.; Huang, J. L.; Chen, J.; Hu, Y. W.; Hu, X. M.; Ren, T. Y.; Zheng, S. H.; Lin, J. D.; Tang, J.; Zheng, L.; Wang, Q., Long non-coding RNA UBE2CP3 promotes tumor metastasis by inducing epithelial-mesenchymal transition in hepatocellular carcinoma. *Oncotarget* **2017,** 8, (39), 65370-65385.
14. Chen, C. S.; Yan, H. L.; Wang, H. J.; Zhou, Y.; Ren, L.; Hou, L. Z.; Yangdan, C. R.; Fan, H. N., *Academic Journal of Second Military Medical University* **2017,** 38, (5), 683-686.
15. Ma, W.; Wang, H.; Jing, W.; Zhou, F.; Chang, L.; Hong, Z.; Liu, H.; Liu, Z.; Yuan, Y., Downregulation of long non-coding RNAs JPX and XIST is associated with the prognosis of hepatocellular carcinoma. *Clin Res Hepatol Gastroenterol* **2017,** 41, (2), 163-170.

16. Qin, Y.; Wu, J.; Ke, Z.; Xu, J., Expression of plasma lncRNA BANCR in hepatocellular carcinoma and its diagnostic and prognostic significance. *International Journal of Clinical and Experimental Medicine* **2017,** 10, (8), 11984-11990.
17. Yuan, W.; Sun, Y.; Liu, L.; Zhou, B.; Wang, S.; Gu, D., Circulating LncRNAs Serve as Diagnostic Markers for Hepatocellular Carcinoma. *Cell Physiol Biochem* **2017,** 44, (1), 125-132.
18. Abd El Gwad, A.; Matboli, M.; El-Tawdi, A.; Habib, E. K.; Shehata, H.; Ibrahim, D.; Tash, F., Role of exosomal competing endogenous RNA in patients with hepatocellular carcinoma. *J Cell Biochem* **2018,** 119, (10), 8600-8610.
19. Gao, S.; Xu, X.; Wang, Y.; Zhang, W.; Wang, X., Diagnostic utility of plasma lncRNA small nucleolar RNA host gene 1 in patients with hepatocellular carcinoma. *Mol Med Rep* **2018,** 18, (3), 3305-3313.
20. Gong, L.; Zhu, X.; Zhang, W.; Ni, H.; Yin, W.; Fu, M., Plasma lncrnas acting as fingerprints in predicting hepatocellular carcinoma from hbv positive chronic hepatitis. *International Journal of Clinical and Experimental Medicine* **2018,** 11, (11), 12088-12096.
21. Gramantieri, L.; Baglioni, M.; Fornari, F.; Laginestra, M. A.; Ferracin, M.; Indio, V.; Ravaioli, M.; Cescon, M.; De Pace, V.; Leoni, S.; Coada, C. A.; Negrini, M.; Bolondi, L.; Giovannini, C., LncRNAs as novel players in hepatocellular carcinoma recurrence. *Oncotarget* **2018,** 9, (80), 35085-35099.
22. Huang, L.; Li, X.; Gao, W., Long non-coding RNA linc-ITGB1 promotes cell proliferation, migration, and invasion in human hepatoma carcinoma by up-regulating ROCK1. *Biosci Rep* **2018,** 38, (5).
23. Li, B.; Mao, R.; Liu, C.; Zhang, W.; Tang, Y.; Guo, Z., LncRNA FAL1 promotes cell proliferation and migration by acting as a CeRNA of miR-1236 in hepatocellular carcinoma cells. *Life Sci* **2018,** 197, 122-129.
24. Luo, P.; Liang, C.; Zhang, X.; Liu, X.; Wang, Y.; Wu, M.; Feng, X.; Tu, J., Identification of long non-coding RNA ZFAS1 as a novel biomarker for diagnosis of HCC. *Biosci Rep* **2018,** 38, (4).
25. Sun, L.; Su, Y.; Liu, X.; Xu, M.; Chen, X.; Zhu, Y.; Guo, Z.; Bai, T.; Dong, L.; Wei, C.; Cai, X.; He, B.; Pan, Y.; Sun, H.; Wang, S., Serum and exosome long non coding RNAs as potential biomarkers for hepatocellular carcinoma. *Journal of Cancer* **2018,** 9, (15), 2631-2639.
26. Wang, Y.; Jing, W.; Ma, W.; Liang, C.; Chai, H.; Tu, J., Down-regulation of long non-coding RNA GAS5-AS1 and its prognostic and diagnostic significance in hepatocellular carcinoma. *Cancer Biomark* **2018,** 22, (2), 227-236.
27. Wang, Z. F.; Hu, R.; Pang, J. M.; Zhang, G. Z.; Yan, W.; Li, Z. N., Serum long noncoding rna lrb1 as a potential biomarker for predicting the diagnosis and prognosis of human hepatocellular carcinoma. *Oncology Letters* **2018,** 16, (2), 1593-1601.
28. Xie, Z.; Zhou, F.; Yang, Y.; Li, L.; Lei, Y.; Lin, X.; Li, H.; Pan, X.; Chen, J.; Wang, G.; Liu, H.; Jiang, J.; Wu, B., Lnc-PCDH9-13:1 Is a Hypersensitive and Specific Biomarker for Early Hepatocellular Carcinoma. *EBioMedicine* **2018,** 33, 57-67.
29. Xu, H.; Chen, Y.; Dong, X.; Wang, X., Serum Exosomal Long Noncoding RNAs ENSG00000258332.1 and LINC00635 for the Diagnosis and Prognosis of Hepatocellular Carcinoma. *Cancer Epidemiol Biomarkers Prev* **2018,** 27, (6), 710-716.

30. Zeng, B.; Lin, Z.; Ye, H.; Cheng, D.; Zhang, G.; Zhou, J.; Huang, Z.; Wang, M.; Cai, C.; Zeng, J.; Tang, C.; Liu, J., Upregulation of LncDQ is Associated with Poor Prognosis and Promotes Tumor Progression via Epigenetic Regulation of the EMT Pathway in HCC. *Cell Physiol Biochem* **2018,** 46, (3), 1122-1133.
31. Zhang, J.; Zhang, D.; Zhao, Q.; Qi, J.; Li, X.; Qin, C., A distinctively expressed long noncoding RNA, RP11-466I1.1, may serve as a prognostic biomarker in hepatocellular carcinoma. *Cancer Medicine* **2018,** 7, (7), 2960-2968.
32. Zheng, Z. K.; Pang, C.; Yang, Y.; Duan, Q.; Zhang, J.; Liu, W. C., Serum long noncoding RNA urothelial carcinoma-associated 1: A novel biomarker for diagnosis and prognosis of hepatocellular carcinoma. *J Int Med Res* **2018,** 46, (1), 348-356.
33. Cao, S. Q.; Zheng, H.; Sun, B. C.; Wang, Z. L.; Liu, T.; Guo, D. H.; Shen, Z. Y., Long non-coding rna highly up-regulated in liver cancer promotes exosome secretion. *World Journal of Gastroenterology* **2019,** 25, (35), 5283-5299.
34. Chao, Y.; Zhou, D., lncRNA-D16366 Is a Potential Biomarker for Diagnosis and Prognosis of Hepatocellular Carcinoma. *Med Sci Monit* **2019,** 25, 6581-6586.
35. Dong, H.; Zhang, Y.; Xu, Y.; Ma, R.; Liu, L.; Luo, C.; Jiang, W., Downregulation of long non-coding RNA MEG3 promotes proliferation, migration, and invasion of human hepatocellular carcinoma cells by upregulating TGF-β1. *Acta Biochim Biophys Sin (Shanghai)* **2019,** 51, (6), 645-652.
36. Habieb, A.; Matboli, M.; El-Tayeb, H.; El-Asmar, F., Potential role of lncRNA-TSIX, miR-548-a-3p, and SOGA1 mRNA in the diagnosis of hepatocellular carcinoma. *Mol Biol Rep* **2019,** 46, (4), 4581-4590.
37. Motawi, T. M. K.; El-Maraghy, S. A.; Sabry, D.; Mehana, N. A., The expression of long non coding RNA genes is associated with expression with polymorphisms of HULC rs7763881 and MALAT1 rs619586 in hepatocellular carcinoma and HBV Egyptian patients. *J Cell Biochem* **2019,** 120, (9), 14645-14656.
38. Qi, J.; Li, J.; Sun, J.; Li, C., Long Non-Coding RNA IGF2AS in Serum may be a Biomarker for Diagnosis of Hepatitis B Virus-Related Hepatocellular Carcinoma. *Clin Lab* **2019,** 65, (12).
39. Refai, N. S.; Louka, M. L.; Halim, H. Y.; Montasser, I., Long non-coding RNAs (CASC2 and TUG1) in hepatocellular carcinoma: Clinical significance. *J Gene Med* **2019,** 21, (9), e3112.
40. Shaker, O. G.; Abdelwahed, M. Y.; Ahmed, N. A.; Hassan, E. A.; Ahmed, T. I.; Abousarie, M. A.; Ayoub, S. E., Evaluation of serum long noncoding RNA NEAT and MiR-129-5p in hepatocellular carcinoma. *IUBMB Life* **2019,** 71, (10), 1571-1578.
41. Wu, F.; Wang, C. X.; Tian, H., Expression and clinical significance of LncRNA ZFAS1 in serum exosomes of patients with hepatocellular carcinoma. *Chinese Journal of Cancer Prevention and Treatment* **2019,** 26, (12), 849-854.
42. Xu, X.; Gu, J.; Ding, X.; Ge, G.; Zang, X.; Ji, R.; Shao, M.; Mao, Z.; Zhang, Y.; Zhang, J.; Mao, F.; Qian, H.; Xu, W.; Cai, H.; Wang, F.; Zhang, X., LINC00978 promotes the progression of hepatocellular carcinoma by regulating EZH2-mediated silencing of p21 and E-cadherin expression. *Cell Death Dis* **2019,** 10, (10), 752.
43. Zeng, Y. L.; Guo, Z. Y.; Su, H. Z.; Zhong, F. D.; Jiang, K. Q.; Yuan, G. D., Diagnostic and prognostic value of lncRNA cancer susceptibility candidate 9 in hepatocellular carcinoma. *World J Gastroenterol* **2019,** 25, (48), 6902-6915.

44. Zeng, Z.; Dong, J.; Li, Y.; Dong, Z.; Liu, Z.; Huang, J.; Wang, Y.; Zhen, Y.; Lu, Y., The expression level and clinical significance of lncRNA X91348 in hepatocellular carcinoma. *Artif Cells Nanomed Biotechnol* **2019,** 47, (1), 3067-3071.
45. Fang, G.; Wang, J.; Sun, X.; Xu, R.; Zhao, X.; Shao, L.; Sun, C.; Wang, Y., LncRNA MAGI2-AS3 is downregulated in the distant recurrence of hepatocellular carcinoma after surgical resection and affects migration and invasion via ROCK2. *Ann Hepatol* **2020,** 19, (5), 535-540.
46. Huang, J.; Zheng, Y.; Xiao, X.; Liu, C.; Lin, J.; Zheng, S.; Yang, B.; Ou, Q., A Circulating Long Noncoding RNA Panel Serves as a Diagnostic Marker for Hepatocellular Carcinoma. *Dis Markers* **2020,** 2020, 5417598.
47. Huang, X.; Sun, L.; Wen, S.; Deng, D.; Wan, F.; He, X.; Tian, L.; Liang, L.; Wei, C.; Gao, K.; Fu, Q.; Li, Y.; Jiang, J.; Zhai, R.; He, M., RNA sequencing of plasma exosomes revealed novel functional long noncoding RNAs in hepatocellular carcinoma. *Cancer Sci* **2020,** 111, (9), 3338-3349.
48. Jiang, L.; He, Y.; Shen, G.; Ni, J.; Xia, Z.; Liu, H.; Cao, Y.; Li, X., lncRNA HAND2-AS1 mediates the downregulation of ROCK2 in hepatocellular carcinoma and inhibits cancer cell proliferation, migration and invasion. *Mol Med Rep* **2020,** 21, (3), 1304-1309.
49. Kim, S. S.; Baek, G. O.; Ahn, H. R.; Sung, S.; Seo, C. W.; Cho, H. J.; Nam, S. W.; Cheong, J. Y.; Eun, J. W., Serum small extracellular vesicle-derived LINC00853 as a novel diagnostic marker for early hepatocellular carcinoma. *Mol Oncol* **2020,** 14, (10), 2646-2659.
50. Li, Y.; Li, G.; Chen, X.; Huang, H.; Liao, L.; Yuan, T.; Deng, S., A novel lncrna nonhsat053785 acts as an independent risk factor for intrahepatic metastasis of hepatocellular carcinoma. *OncoTargets and Therapy* **2020,** 13, 5455-5466.
51. Lu, Y.; Duan, Y.; Xu, Q.; Zhang, L.; Chen, W.; Qu, Z.; Wu, B.; Liu, W.; Shi, L.; Wu, D.; Yang, Y.; Sun, D.; Chen, X., Circulating exosome-derived bona fide long non-coding RNAs predicting the occurrence and metastasis of hepatocellular carcinoma. *J Cell Mol Med* **2020,** 24, (2), 1311-1318.
52. Matboli, M.; Labib, M. E.; Nasser, H. E. T.; El-Tawdi, A. H. F.; Habib, E. K.; Ali-Labib, R., Exosomal miR-1298 and lncRNA-rp11-583F2.2 expression in hepatocellular carcinoma. *Current Genomics* **2020,** 21, (1), 37-46.
53. Mohyeldeen, M.; Ibrahim, S.; Shaker, O.; Helmy, H., Serum expression and diagnostic potential of long non-coding RNAs NEAT1 and TUG1 in viral hepatitis C and viral hepatitis C-associated hepatocellular carcinoma. *Clin Biochem* **2020,** 84, 38-44.
54. Qin, S. J.; Zhou, H. Z.; Xu, N. S.; Yang, H. C.; Chen, P. X., The Diagnostic Value of Serum ST8SIA6-AS1 as Biomarker in Hepatocellular Carcinoma. *Clin Lab* **2020,** 66, (12).
55. Roshdy, F.; Farag, M. M. S.; El-Ahwany, E.; Mahmode, O.; Mousa, A. A.; El Talkawy, M.; Essawy, F., Long non-coding RNA HOTAIR and HOTTIP as potential biomarkers for hepatitis C virus genotype 4-induced hepatocellular carcinoma. *Egyptian Journal of Medical Human Genetics* **2020,** 21, (1).
56. Shaker, O. G.; Khairy, A. M.; Ali, R. M. M.; Badr, A. M., miRNA-141 and its target long non-coding RNA HOTAIR as diagnostic marker in hepatocellular carcinoma on top of hepatitis C virus. *Gene Reports* **2020,** 21.

57. Song, W.; Wu, J.; Wu, L.; Zhao, H., Signifi cance of serum lncRNA-PVT1 expression in diagnosis and prognosis of hepatocellular carcinoma. *Journal of Practical Oncology* **2020,** 35, (4), 317-321.
58. Yao, Z.; Jia, C.; Tai, Y.; Liang, H.; Zhong, Z.; Xiong, Z.; Deng, M.; Zhang, Q., Serum exosomal long noncoding RNAs lnc-FAM72D-3 and lnc-EPC1-4 as diagnostic biomarkers for hepatocellular carcinoma. *Aging (Albany NY)* **2020,** 12, (12), 11843-11863.
59. Kim, S. S.; Baek, G. O.; Son, J. A.; Ahn, H. R.; Yoon, M. K.; Cho, H. J.; Yoon, J. H.; Nam, S. W.; Cheong, J. Y.; Eun, J. W., Early detection of hepatocellular carcinoma via liquid biopsy: panel of small extracellular vesicle-derived long noncoding RNAs identified as markers. *Mol Oncol* **2021,** 15, (10), 2715-2731.
60. Tian, Q.; Yan, X.; Yang, L.; Liu, Z.; Yuan, Z.; Zhang, Y., Long non-coding RNA BACE1-AS plays an oncogenic role in hepatocellular carcinoma cells through miR-214-3p/APLN axis. *Acta Biochim Biophys Sin (Shanghai)* **2021,** 53, (11), 1538-1546.
61. Wang, T.; Zhu, H.; Xiao, M.; Zhou, S., Serum exosomal long noncoding RNA CRNDE as a prognostic biomarker for hepatocellular carcinoma. *J Clin Lab Anal* **2021,** 35, (11), e23959.
62. You, L. N.; Tai, Q. W.; Xu, L.; Hao, Y.; Guo, W. J.; Zhang, Q.; Tong, Q.; Zhang, H.; Huang, W. K., Exosomal LINC00161 promotes angiogenesis and metastasis via regulating miR-590-3p/ROCK axis in hepatocellular carcinoma. *Cancer Gene Ther* **2021,** 28, (6), 719-736.
63. Zhu, X.; Feng, Y.; He, D.; Wang, Z.; Huang, F.; Tu, J., Clinical Value and Underlying Mechanisms of Upregulated LINC00485 in Hepatocellular Carcinoma. *Frontiers in Oncology* **2021,** 11.
64. Ali, M. A.; Shaker, O. G.; Ezzat, E. M.; Eid, H. M.; Ali, D. Y.; Hassan, E. A.; Elsayed, D. H.; Abozaid, E. R.; Abdelaleem, O. O., Serum lncRNAs, NBAT-1, and FOXCUT signature in hepatocellular carcinoma developed on top of chronic hepatitis C. *Molecular Carcinogenesis* **2022**.
65. Bongolo, C. C.; Thokerunga, E.; Fidele, N. B.; Souraka, T. D. M.; Kisembo, P.; Rugera, S. P.; Worley, P. F.; Tu, J. C., Upregulation of the long non-coding RNA, LIPCAR promotes proliferation, migration, and metastasis of hepatocellular carcinoma. *Cancer Biomark* **2022,** 35, (3), 245-256.
66. Chen, J.; Tang, D.; Li, H.; Zhang, P., Expression changes of serum LINC00941 and LINC00514 in HBV infection-related liver diseases and their potential application values. *J Clin Lab Anal* **2022,** 36, (1), e24143.
67. Chen, Y.; Zhang, J.; Liu, J.; Wang, J.; Shi, C.; Lu, L.; Cheng, X.; Niu, G.; Zhang, S., The Expression and Clinical Significance of PCNAP1 in Hepatocellular Carcinoma Patients. *J Immunol Res* **2022,** 2022, 1817694.
68. El-Shendidi, A.; Ghazala, R.; Hassouna, E., Circulating HOTAIR potentially predicts hepatocellular carcinoma in cirrhotic liver and prefigures the tumor stage. *Clinical and Experimental Hepatology* **2022,** 8, (2), 139-146.
69. Fu, P.; Gong, B.; Li, H.; Luo, Q.; Huang, Z.; Shan, R.; Li, J.; Yan, S., Combined identification of three lncRNAs in serum as effective diagnostic and prognostic biomarkers for hepatitis B virus-related hepatocellular carcinoma. *Int J Cancer* **2022,** 151, (10), 1824-1834.

70. Gong, A.; Luo, X.; Tan, Y.; Chen, H.; Luo, G., High expression of C10orf91 and LINC01224 in hepatocellular carcinoma and poor prognosis. *American Journal of Translational Research* **2022,** 14, (4), 2567-2579.
71. Han, Y.; Jiang, W.; Wang, Y.; Zhao, M.; Li, Y.; Ren, L., Serum long non-coding RNA SCARNA10 serves as a potential diagnostic biomarker for hepatocellular carcinoma. *BMC Cancer* **2022,** 22, (1), 431.
72. Lou, Z. H.; Xu, K. Y.; Qiao, L.; Su, X. Q.; Ou-Yang, Y.; Miao, L. B.; Liu, F.; Wang, Y.; Fu, A.; Ren, X. H.; Huang, J. S.; Li, Z. Y.; Bao, J. F., Diagnostic Potential of the Serum lncRNAs HOTAIR, BRM and ICR for Hepatocellular Carcinoma. *Front Biosci (Landmark Ed)* **2022,** 27, (9), 264.
73. Manganelli, M.; Grossi, I.; Corsi, J.; D'Agostino, V. G.; Jurikova, K.; Cusanelli, E.; Molfino, S.; Portolani, N.; Salvi, A.; De Petro, G., Expression of Cellular and Extracellular TERRA, TERC and TERT in Hepatocellular Carcinoma. *Int J Mol Sci* **2022,** 23, (11).
74. Mo, C.; Wu, J.; Sui, J.; Deng, Y.; Li, M.; Cao, Z.; Hu, Z.; Huang, J.; Li, S., Long non-coding RNA LINC01793 as a potential diagnostic biomarker of hepatitis B virus-related hepatocellular carcinoma. *Clin Biochem* **2022,** 108, 56-62.
75. Mohammed, S. R.; Shaker, O. G.; Mohamed, M. M.; Abdelhafez Mostafa, M. N.; Gaber, S. N.; Ali, D. Y.; Hussein, H. A.; Elebiary, A. M.; Abonar, A. A.; Eid, H. M.; El Sayed Ali, H. S., The emerging role of lncRNA MEG3 and MEG3 rs7158663 in hepatocellular carcinoma. *Eur Rev Med Pharmacol Sci* **2022,** 26, (1), 11-21.
76. Yao, J.; Hua, X.; Shi, J.; Hu, X.; Lui, K.; He, K.; Mai, J.; Lan, T.; Lu, M., LncRNA THEMIS2-211, a tumor-originated circulating exosomal biomarker, promotes the growth and metastasis of hepatocellular carcinoma by functioning as a competing endogenous RNA. *Faseb j* **2022,** 36, (4), e22238.
